# Tuberculous Pericarditis in Childhood: A Case Report and a Systematic Literature Review

**DOI:** 10.3390/pathogens13020110

**Published:** 2024-01-26

**Authors:** Laura Venuti, Anna Condemi, Chiara Albano, Giovanni Boncori, Valeria Garbo, Sara Bagarello, Antonio Cascio, Claudia Colomba

**Affiliations:** 1 Department of Health Promotion, Mother and Child Care, Internal Medicine and Medical Specialties “G D’Alessandro”, University of Palermo, 90127 Palermo, Italy; annacondemi96@gmail.com (A.C.); ch.albano27@gmail.com (C.A.); boncori.giovanni@yahoo.it (G.B.); vali.garbo@gmail.com (V.G.); sbagarello@gmail.com (S.B.); claudia.colomba@unipa.it (C.C.); 2 Infectious and Tropical Disease Unit, Sicilian Regional Reference Center for the Fight against AIDS, AOU Policlinico “P. Giaccone”, 90127 Palermo, Italy; antonio.cascio03@unipa.it; 3 Division of Paediatric Infectious Disease, “G. Di Cristina” Hospital, ARNAS Civico Di Cristina Benfratelli, 90127 Palermo, Italy

**Keywords:** tuberculosis, pericarditis, pericardial effusion, tuberculous pericarditis, childhood, pediatric tuberculosis, TST, antitubercular treatment, pericardiocentesis, pericardiectomy

## Abstract

Tuberculous pericarditis (TBP) is an important cause of pericarditis worldwide while being infrequent in childhood, especially in low-TB-incidence countries. We report a case of TBP and provide a systematic review of the literature, conducted by searching PubMed, Scopus, and Cochrane to find cases of TBP in pediatric age published in the English language between the year 1990 and the time of the search. Of the 587 search results obtained, after screening and a backward citation search, 45 studies were selected to be included in this review, accounting for a total of 125 patients. The main signs and symptoms were fever, cough, weight loss, hepatomegaly, dyspnea, and increased jugular venous pressure or jugular vein turgor. A definitive diagnosis of TBP was made in 36 patients, either thanks to microbiological investigations, histological analysis, or both. First-line antitubercular treatment (ATT) was administered in nearly all cases, and 69 children underwent surgical procedures. Only six patients died, and only two died of TBP. TBP in childhood is relatively uncommon, even in high-TB-prevalence countries. Clinical manifestations, often suggestive of right-sided cardiac failure, are subtle, and diagnosis is challenging. TBP has an excellent prognosis in childhood; however, in a significant proportion of cases, invasive surgical procedures are necessary.

## 1. Introduction

Tuberculosis (TB) is currently a global public health concern, with an estimated annual incidence ranging from 9 to 11 million cases worldwide, including 1.2 million cases among children [1,2]. Its reported mortality rate reached approximately 1.6 million deaths in the year 2021 alone, making TB the 13th leading cause of death globally and the primary infective cause of death after COVID-19, surpassing even HIV and AIDS [1,2]. Extrapulmonary tuberculosis (EPTB) accounts for 15–20% of all cases of TB in immunocompetent patients and for more than 50% of cases among HIV-positive people [3].

Tuberculous pericarditis (TBP) is the predominant cardiac manifestation of TB [4], and it is considered rare in low-TB-burden countries while being one of the main causes of pericarditis in TB-endemic regions [5,6]. Occurring in 1% to 4% of pulmonary TB cases [7,8] and accounting for 50–90% of cases of pericarditis, TBP has a substantial impact on cardiovascular death and disability [9,10].

TBP has historically been considered “a disease of adult males” [11], and its occurrence in childhood has been found to be rare [12,13,14,15,16,17].

Both diagnosis and management of TBP remain complex and challenging due to its insidious clinical presentation and the lack of simple, rapid, and readily accessible diagnostic tests [3]. Without timely and appropriate intervention, TBP can lead to severe complications, such as constrictive pericarditis, cardiac tamponade, and even death [10].

We report a rare case of TBP in a previously healthy child and provide a systematic review of the literature to offer a comprehensive overview of the epidemiological and clinical characteristics of pediatric TBP.

## 2. Case Report 

A 12-year-old girl of Italian origin was admitted to the pediatric emergency department in September 2022 with a suspected urinary tract infection. The relatives reported a history of lower-limb edemas, which had been present for several weeks, and an unremarkable, non-productive cough lasting for a couple of days in the absence of chest pain. Acute kidney injury, glomerulonephritis, and nephrotic syndrome were ruled out thanks to hematological and biochemical tests, which revealed only a mild elevation in inflammatory markers. 

Relevant findings on physical examination were firm edema affecting the lower limbs up to the knees, subcutaneous edema in the abdominal region, a distended abdomen with a liver protrusion of 2–3 cm beyond the costal margin, diminished breath sounds at the left lung base, jugular vein distention, and tachycardia.

Echocardiography showed a normal-sized left ventricle with a 52–55% ejection fraction, interventricular septal dyskinesia, dilation of both atria, and mild-to-moderate mitral regurgitation. Of note, the right ventricle appeared reduced in size due to extrinsic compression, the inferior vena cava was dilated, and the pericardium appeared refractive in the anterior region, free of effusion.

**Figure 1:**
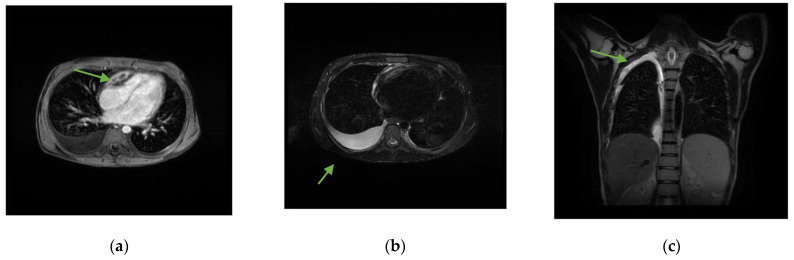
Chest MRI with and without contrast medium. (**a**) Solid tissue, with some central hypointense areas on T1 and T2 sequences without contrast enhancement, as from fibrosis, located in the upper anterior mediastinal area (arrow) of approximately 6.5 × 2 cm, adherent to the pericardium, which appears thickened in correspondence with the right heart sections, with a consequent compressive effect on the ventricle. (**b**) Right pleural effusion (arrow) extended to the apex in the supine position (**c**) with a maximum thickness of approximately 3 cm.

Further diagnostic investigation, conducted through chest and abdominal CT scans and a subsequent MRI (Figure 1), revealed the presence of a mediastinal mass adhering to the pericardium and compressing the right ventricle (RV), mediastinal and mesenteric lymphadenomegaly, hepatosplenomegaly, pleural effusion, and ascitic fluid.

Due to suspicion of a mediastinal neoplastic lesion, the patient was transferred to the pediatric oncohematology department, where a bone marrow aspiration yielded no neoplastic cells, and a PET scan revealed radioisotope uptake in the mediastinal mass and mediastinal and mesenteric lymph nodes. Histopathological examination of the mediastinal mass revealed the presence of granulomatous lesions evolving towards necrosis.

In light of histopathological findings and after a consultation with an infectious disease specialist, a tuberculin skin test and an IGRA test (Quantiferon) were performed and had positive results. Upon admission to the infectious disease department, the patient was afebrile with persistent signs of right-sided heart failure. A serial monitoring process was initiated, consisting of repeated echocardiography, Holter ECG, and specialized cardiological, cardiosurgical, and pneumological consultations. 

Although the Ziehl–Neelsen stain and the polymerase chain reaction (PCR) for MTB essay performed on urine and sputum samples had a negative outcome, a four-drug anti-TB regimen with isoniazid, rifampicin, ethambutol, and pyrazinamide was started in association with steroids (prednisone, 10 mg three times per day). Unfortunately, diagnostic imaging follow-up at one month failed to show improvement, and a decompressive pericardiectomy was ultimately necessary.

During surgery, a strong pericardial–epicardial adhesion was observed, and a pocket of caseous necrosis underlying the atrioventricular sulcus was detected. The caseous material was evacuated by opening the anterior leaflet of the pocket and collected to perform a culture for MTB, which confirmed the suspected diagnosis of constrictive TBP.

As of today, the patient is being monitored and followed through convalescence and rehabilitation while completing the antitubercular treatment.

## 3. Materials and Methods 

We conducted a systematic review using PubMed, Scopus, and Cochrane to find studies that reported cases of TBP in children in all countries and regions. Our search string was initially developed for PubMed to include all potential pediatric cases of TBP and then appropriately translated to Scopus and Cochrane (Additional File S1). We checked the reference lists of the included papers to find additional studies on pediatric TBP, thus performing a backward citation search.

First, an overall screening of articles by title and abstract was carried out, and then a full-text assessment was conducted, followed by a selection of pertinent papers based on the eligibility criteria described below. No automation tools were used.

Inclusion criteria—The patients included were children (age 0 to 18 years old) with probable or confirmed TBP (Additional File S2). Only studies published between 1990 and the time of the search (July 2023) and involving direct observation, examination, or analysis of patients with TBP in the pediatric population were considered eligible for inclusion in this systematic review. Study types included were case reports of children with TBP, case reports of children with particular conditions who also had TBP, case series on TBP in children (in three instances [18,19,20]), and pediatric case series with a main focus other than TBP, which also included cases of TBP [21].

Our focus of interest was TBP in pediatric age in all its aspects: epidemiology, clinical manifestations, diagnosis, treatment, and outcome. However, studies that did not include information on all of these aspects were also included, clearly stating when data were missing and which type of information was not available.

Exclusion criteria—We excluded studies belonging to one of the following categories: articles published before the year 1990; articles not available in the English language; articles that were primarily theoretical or non-clinical, such as editorials or general lectures that did not provide specific patient-centered data; articles unrelated to TBP; articles reporting on TBP in adults; articles which included one or more cases of TBP in children but where it was not possible to isolate and extract relevant data specific to these cases (the authors reported on data such as clinical manifestations collectively, including the entire population under observation); articles where cases of TBP and children were included but it was unclear whether the cases of TBP affected the children in the study.

Data extraction and analysis—Data were extracted and analyzed using Microsoft Excel 2018 spreadsheets, which included several variables, as reported in Additional File S3. 

This systematic review was performed following the PRISMA guidelines (Reporting Items for Systematic Reviews and Meta-Analyses) [22].

## 4. Results

**Figure 2:**
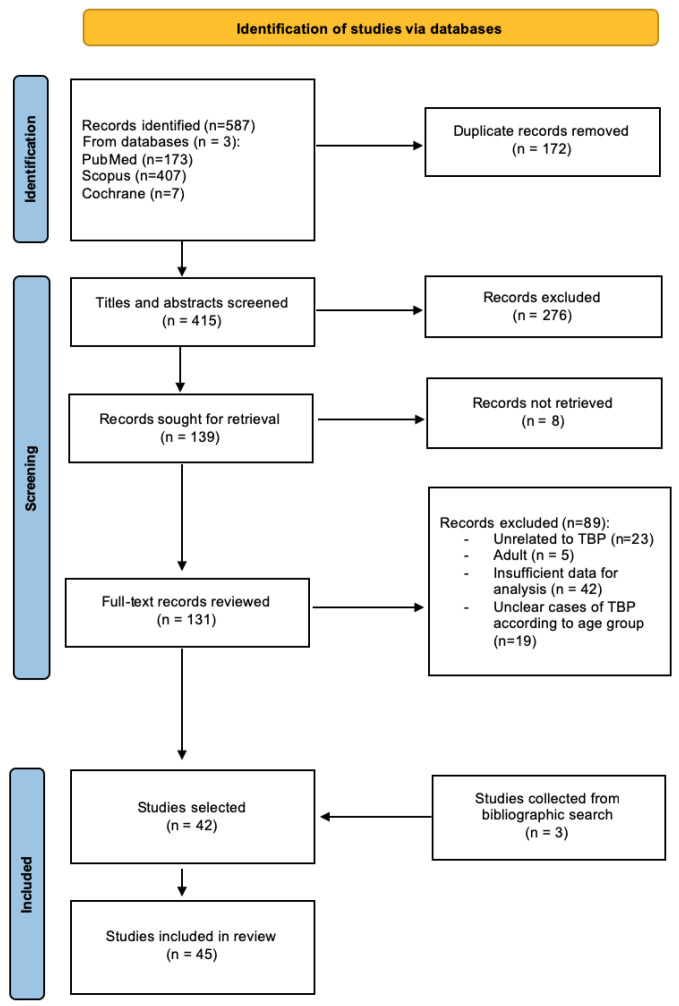
Flow chart describing the study identification and selection process.

Study selection process—The search strategy initially resulted in 587 publications: 173 from PubMed, 407 from Scopus, and 7 from Cochrane databases. After eliminating 172 duplicate records, a total of 415 articles underwent initial title and abstract screening. Subsequently, 276 records were excluded based on predefined criteria. The remaining 139 records were selected for full-text retrieval, of which eight were not successfully retrieved. Following a comprehensive review of 131 full-text records, an additional 89 records were excluded for various reasons, as depicted in Figure 2. Furthermore, a backward citation search contributed to the identification of three additional relevant studies. Ultimately, this systematic review comprises a total of 45 studies [11,18,19,20,21,23,24,25,26,27,28,29,30,31,32,33,34,35,36,37,38,39,40,41,42,43,44,45,46,47,48,49,50,51,52,53,54,55,56,57,58,59,60,61,62].

Relevant data for each study, such as epidemiological and clinical characteristics, diagnostic procedures, treatment, and outcome, are collectively reported in Table 1.

Epidemiology—The studies incorporated in this analysis encompassed 26 distinct countries (Additional File S4) and collectively involved 125 patients. Given dataset limitations, we presumed that the patients’ origins aligned with the countries where the studies were conducted. In one instance, discerning the study’s host country proved challenging due to the multicentric nature of the research, which involved multiple study groups from various nations [29].

Among the total of 125 cases examined in this review, information on sex was missing in three cases. Of the remaining 122 cases, 55 were identified as female (45%). The overall median age recorded was 10 years.

Out of the complete cohort of cases included in this review, a notable 80% originated from countries categorized as high TB-burden nations, according to the classification reported in the World Health Organization’s latest global list [63] (based on 2021 data and subject to revisions in 2025). Specifically, 76 children (61%) originated from South Africa.

Clinical manifestations—The most significant signs and symptoms, along with their respective frequencies, have been compiled in Table 2 using the available data from 123 cases. However, for two cases where the information is generic (e.g., ‘signs and symptoms of constrictive pericarditis’ or ‘cardiac tamponade’), specific details are not known.

Notably, in 47 cases (38%), anemia was present.

In 18 of 120 cases (15%) where the type of TBP was reported, this was identified as constrictive TBP, while the rest were of either an effusive nature with varying levels of fluid accumulation or demonstrated a mixed pattern reflecting both effusive and constrictive characteristics. Furthermore, in 53 out of the 120 cases (44%), either “features of cardiac tamponade,” impending, or full-blown cardiac tamponade were reported.

Interestingly, three peculiar forms of TBP were found that do not fall neatly into the traditional classification of pediatric TBP: a cystic form (pericardial tubercular cyst [50]), an isolated calcification that did not cause constriction due to the localized nature of the lesion [48], and an effusive form with large masses floating in the pericardial fluid [41].

In 53 cases (42%), TBP represented the main manifestation of TB, while in the remaining cases, other organs were affected, as depicted in Table 3. In three instances, TB abscesses were identified, including a paraspinal abscess, a chest wall abscess, and an abscess adjacent to the right ventricle.

Diagnosis—In 61 out of 118 cases (52%) where information regarding microbiological investigations was available, MTB was confirmed thanks to tests such as PCR, microscopy with acid-fast bacilli (AFB) coloration, or culture. These tests were performed on different types of biological samples, namely: sputum, pleural fluid, gastric aspirate or washing, pericardial fluid or tissue, ascitic fluid, lymph node aspirate or biopsy, cerebrospinal fluid, bone marrow aspirate, or pus drainage.

In one patient, fluorescence microscopy with auramine O staining was performed on a pericardial biopsy and yielded a positive result [58]. In the same patient, kinyoun coloration for AFB on pericardial tissue and PCR on pericardial fluid were also positive for MTB.

In 21 patients (18%), in particular, MTB was confirmed on pericardial fluid or tissue biopsy, hence meeting the criteria for certain diagnoses of TBP.

In 22 patients, reported histological findings were suggestive of MTB infection. In three cases, histological reports were nonspecific (‘fibrosis,’ ‘chronic inflammation,’ ‘nonspecific evidence of inflammatory cells’). In the remaining cases, no information regarding histological investigations was reported; we assume that in most of these cases, a biopsy was not performed. Histology was performed on biopsy or surgical specimens or, in one case, on an autoptic sample.

Overall, a certain diagnosis was made in 36 patients (29%), either through histological analysis, microbiological confirmation, or both.

In 19 cases, the authors reported the presence of elevated adenosine deaminase (ADA) levels in pericardial fluid, indicating mycobacterial infection. In a case series of 44 patients [18], ADA levels were measured in 22 cases, out of which 14 were higher than 35 U/L. In the remaining five cases where elevated ADA levels were reported, these ranged from 46 to 110 U/L (46–52–90–106–110). In two cases, elevated ADA levels in fluids other than pericardial fluid were reported (ascitic fluid and pleural fluid).

In 84 patients (67%), TST/IGRA results were reported: 60 of these (71%) were positive and 24 (29%) were negative. In 80 cases (64%), cardiomegaly was reported on CXR.

In 115 patients (92%), pericarditis was confirmed through echocardiographic findings, which showed variable amounts of effusions or constrictive pericarditis patterns (details are described in Table 1). In the remaining ten cases, either imaging findings were not reported (six cases) or pericardial involvement was confirmed through other imaging techniques, e.g., CT or CXR (four cases).

Treatment—In 122 out of 124 cases (98%) where data were available, first-line antitubercular treatment was administered. In two cases, treatment was not administered, either due to parental disagreement or because TBP was an autoptic finding. In eight cases (6%), second-line antitubercular treatment was administered due to findings consistent with drug-resistant MTB. In 67 cases (54%), steroids were administered.

Overall, 69 children (55%) underwent some type of invasive procedure. In particular, 22 (18%) underwent pericardiectomy; 20 (16%) underwent pericardiotomy, pericardiostomy, or pericardial window procedures; and 48 (38%) underwent pericardiocentesis (sometimes repeated), of which five were emergency pericardiocentesis. In one case, a pericardial mass was surgically removed, and in another case, a tuberculous pericardial cyst was removed [37,50]. In other cases, surgery was performed to treat conditions other than pericarditis, such as a recurrent subaortic aneurysm [46], a recurrent LV aneurysm [43], or a pulmonary occlusion caused by tubercular lymphadenopathy [54].

In 108 of 114 cases where the outcome was documented (95%), treatment led to an improved clinical picture; six (5%) patients died, and 11 patients (9% of the cohort) were lost to follow-up. Of the deceased patients, one, with Takayasu arteritis, died of a cause other than TBP; one died because he did not receive treatment; one, with meningitis, died of an unspecified cause; one died of ventricular fibrillation after recovering from TBP; one died because of disseminated TB; and one case is an autoptic report [25] of TBP, which is what caused the patient’s sudden death.

## 5. Discussion

TBP is certainly rare in low-TB-burden countries, and different authors underline its relative infrequency in pediatric age, even in high-TB-burden countries [19,34], citing in particular two studies. The first is a case series [18] (included in this review) conducted in South Africa where, over a six-year period, only 44 children were diagnosed with TBP in an institution where TB is the major cause of pericardial disease. The second [64] is a more recent study conducted in India, where TBP was diagnosed only in 13 children over a period longer than three years at a tertiary care center. Moreover, a retrospective study on pediatric TB covering a period of 20 years [65], conducted in two tertiary hospitals in Rome, found only one case of TBP out of a total of 214 patients affected by both pulmonary and extrapulmonary TB. Additionally, a case series [21] conducted in Greece over the span of 16 years found only one case of TBP out of 102 children with EPTB.

Nevertheless, TB remains an important cause of pericardial effusion in high-TB-burden regions, accounting for an estimated occurrence of pericarditis in 1% to 4% of pediatric TB cases [66]. An overwhelming majority (80%) of the cases collected in this review were observed in high-TB-prevalence regions. This albeit staggering proportion might be the result of an underestimation, considering that some of the children treated in low-incidence regions, assumed to be from the same area where they were treated, might have been originating from high-TB-burden countries. These facts highlight the impact of the social determinants of health on a child’s risk of contracting TBP.

The progression of tuberculous pericarditis consists of four stages [5,10,67]:

- In the dry stage, when the bacillus reaches the pericardium via the retrograde lymphatic spread, hematogenous dissemination or contiguity from surrounding tissues and a poorly organized lymphomonocytic inflammatory infiltrate appear. This stage, characterized by clinical manifestations of acute pericarditis such as chest pain, pericardial friction rub, and ST elevation in the absence of effusion, is rarely observed in TBP in children.
- An effusive stage, the most commonly observed, can manifest clinically with signs and symptoms of heart failure or cardiac tamponade due to a conspicuous effusion, or it can present in mixed form, with both a compressive effusion and a visceral constriction present at the same time.
- An adsorptive stage is characterized by reabsorption of the pericardial effusion, organization of the infiltrate to form granulomas with caseous necrosis, and thickening of the pericardium due to fibrin and collagen deposition. In this stage, signs and symptoms are those of constrictive pericarditis, although a dense fluid can still be found through echocardiography or imaging.
- A constrictive stage, when there is no residual fluid and fibrosis of the visceral and parietal pericardial layers, leads to the formation of a fibrocalcific stratum that encases the cardiac chambers, resulting in the classic presentation of constrictive pericarditis.

Pediatric TBP manifests with signs and symptoms of infection, such as fever, cough, weight loss, and right-sided heart failure, chiefly hepatomegaly and jugular vein turgor. However, signs and symptoms specific to pericarditis, such as chest pain, friction rub, and muffled heart sounds, are infrequent. Therefore, clinical diagnosis is challenging. Moreover, complications such as constrictive pericarditis and cardiac tamponade are not infrequent, thus highlighting the importance of timely diagnosis.

In this review, comorbidities were observed in 28 cases (Additional File S5). Notably, ten children were HIV positive, and four children were subjected to immune-suppressive treatments for different autoimmune disorders, such as linear IgA bullous dermatosis (LABD), juvenile idiopathic arthritis (JIA), Chron’s disease, primary sclerosing cholangitis, and ulcerative colitis). However, in the vast majority of cases, TBP was not correlated with immune suppression. In three instances, immune reconstitution inflammatory syndrome (IRIS) was observed. In an HIV-positive child [19], IRIS manifested after antiretroviral therapy (ART) and did not worsen TBP. A 3-year-old HIV-positive child [45] was reported to have paradoxical IRIS as he presented with disseminated TB (including TBP) and developed chylous ascites and abdominal lymphadenopathy only after initiation of highly active antiretroviral therapy (HAART). In the third case [29], the author reported on TBP within the context of probable IRIS. Therefore, in the first two cases, TBP was already present when IRIS manifested, and the syndrome was a consequence of antiviral treatment. Conversely, in the third case, TBP was likely a manifestation of IRIS, which was triggered by the antitubercular treatment.

In 11 cases (9%), malnutrition was reported by the authors, representing a risk factor in the development of TBP.

Ten children had HIV, nine of which were observed in a case series [19] conducted by Obihara et al., who found that clinical presentation, TB severity, rates of extrapulmonary TB (other than TBP), and large effusions were similar in HIV-positive and HIV-negative children with TBP.

Laboratory diagnosis of TBP in childhood, like clinical diagnosis, is also complex for several reasons. To begin with, TBP can be and often is the sole manifestation of TB, which raises the issues of clinical suspicion and the obtainability of biological samples through non-invasive procedures. In the context of this review, despite the fact that the presence of MBT was confirmed in approximately half of the cases, it was found in pericardial fluid or tissue in merely 21 cases, or roughly one-third of the microbiologically confirmed cases. Additionally, despite the fact that documentation of negative microbiological results was not frequently provided, we found that in 35 patients, microbiological tests performed on pericardial fluid yielded false negative results, and in 21 cases, investigations performed on pericardial tissue failed to prove MTB presence. Conversely, in 13 patients, MBT was evidenced through microbiological analysis of pericardial fluid and, in 10 cases, pericardial tissue.

Histological diagnosis is an important means to determine a definitive diagnosis, although biopsy samples are infrequently collected because of a lack of diagnostic suspicion or due to the invasive nature of the procedure.

A certain diagnosis is defined as the confirmed presence of MTB in the pericardium (fluid or tissue) and/or histological findings indicating tuberculous infection (such as granulomas, chronic inflammatory infiltrates with histiocytes, and fibrosis). A probable diagnosis of TBP is made when, together with pericarditis, there is evidence of MTB in other bodily sites, elevated ADA levels in pericardial fluid, and epidemiological and clinical characteristics indicative of TB or improvement after antitubercular treatment (ex juvantibus criterion) [5,18,19].

Given the challenges involved in definitively diagnosing TBP, there is a need for more accurate and less invasive methods for diagnosing TBP in children. In this regard, a study found that measuring lysozyme levels in fluids, including pericardial effusions, can help distinguish between tuberculous and non-tuberculous effusions in children [68]. Another study focused on the accuracy of PCR and ADA in the identification of tuberculous effusions in children found that the sensitivity and specificity of PCR were 74% and 88%, while those of ADA levels equal to or superior to 38 IU/L were 81% and 75%, respectively [69]. The authors concluded that combining different tests significantly improves sensitivity. Of note, these procedures do not apply to cases of constrictive pericarditis due to a lack of effusion in the pericardial sac.

In settings with limited resources, a chest X-ray (CXR) is of considerable importance since it raises awareness of pericardial effusion by detecting cardiomegaly (enlarged heart shadow with a globular appearance [70]). Nevertheless, echocardiography stands out as the main imaging tool in the diagnosis of pericarditis, offering crucial insights into its subtype and providing indications about possible causes, such as the presence of fibrinous strands in the pericardial space [71]. Irregularities such as thickening of the pericardium, the presence of an exudative layer, and strands spanning the pericardial space point towards TBP. Notably, pericardial thickening and fibrin strands have a specificity of 94% and 88%, respectively, while exudative coating has a sensitivity of 100% in diagnosing TBP [4,60,72].

Treatment of TBP, which is based on the administration of first-line and sometimes second-line antitubercular medications, is effective, and the prognosis is excellent. Unsurprisingly, the few deaths found in this review were due to lack of treatment, other conditions, other TB manifestations, or unclear causes.

However, it is important to consider that invasive therapeutic procedures are often necessary (as they were in more than half of the cases of this cohort), further highlighting the importance of timely diagnosis. These include pericardial fluid drainage procedures (which, at times, yielded more than 1 L of fluid) and pericardiectomy.

Two randomized controlled trials [73] conducted in Transkei (South Africa), which included but were not exclusive to pediatric patients, showed an advantage in the use of prednisolone for constrictive pericarditis and open drainage and prednisolone for effusive pericarditis. To our knowledge, a similar study focused solely on a pediatric population has never been conducted. However, according to the American Thoracic Society’s consensus statement on TB [74], steroid treatment is indicated for TBP.

We found that of the 18 children who presented with a constrictive form of TBP, 14 underwent pericardiectomy. Of these 14 patients, 7 had not been treated with steroids, 2 received steroids, and in 5 cases, it was not possible to determine whether steroids had been administered. In a case series [18] conducted by Hugo-Hamman et al., all five children who developed constrictive pericarditis were not treated with steroids, and pericardiectomy was necessary in three children who presented with constrictive pericarditis and in two who developed it after drainage. These findings reinforce the idea that treatment of constrictive pericarditis often requires pericardiectomy and that the role of steroids in preventing the necessity of this invasive procedure in childhood might be worth further investigation.

## 6. Conclusions

In conclusion, TBP is more prevalent in areas with a significant TB burden, reflecting the influence of social determinants on a child’s susceptibility to TBP. The diagnostic journey for TBP is complex. While clinical signs and symptoms, including those of right-sided heart failure, can signal TBP, they are often nonspecific and challenging to differentiate from other conditions. Nonetheless, our experience indicates that in the presence of a child with signs and symptoms of cardiac heart failure, diagnostic suspicion of TBP is warranted, even in immunocompetent patients from low-TB-burden countries.

Certain diagnosis relies on histology or microbiological confirmation of MTB in the pericardium, which remains challenging due to the invasiveness of biopsy and the possibility of false-negative results. Echocardiography offers valuable insights not only into features specific to TBP but also indicative of its subtype (constrictive, effusive, or mixed) and can justify the necessity for more invasive diagnostic and therapeutic measures.

Medical treatment, which is based on the administration of antitubercular medications and often steroids, has great efficacy in childhood. Yet, invasive procedures like pericardiectomy remain common, particularly in constrictive TBP. Differently from what has been observed in adults [75], TBP has an excellent prognosis in children when diagnosis is timely and treatment is available. Overall, while strides have been made in understanding and managing TBP in children, continued research efforts are essential to enhance diagnostic accuracy, treatment efficacy, and patient outcomes in this complex clinical entity.

## Figures and Tables

**Table 1:** Overview of relevant data collected from each study. NP “Not performed”; NR “Not reported”; JVP “jugular venous pressure”; JV “jugular veins”; IVC “inferior vena cava”; SVC “superior vena cava”; RV “right ventricle”; LV “left ventricle”; RA “right atrium”; LA “left atrium”; AFB “acid-fast bacilli”; PCR “polymerase chain reaction”; ATT “antitubercular treatment”; HRZE “isoniazid, rifampicin, pyrazinamide, ethambutol”; PAS “para-aminosalicylic acid”; MTBDR “Mycobacterium tuberculosis and drug resistance.”

Epidemiology			Clinical Manifestations		Diagnosis			Treatment And Outcome		
Author, Year, Country	Number of Patients	Sex and Age (years)	Signs and Symptoms	Cardiovascular Signs and Symptoms	Positive Microbiology and/or Elevated ADA	Histology Indicative of MTB	Echocardiographic/US and/or ECG Findings	Medical Treatment	Surgical Treatment	Outcome
Gobir et al. [23] 2022 Nigeria	1	F 11 y	Fever, cough, weight loss, night sweats, lymphadenopathy	Dyspnea, orthopnea, lower limbs pitting edema, ascites and abdominal distention, bilateral neck swelling (bull neck appearance), tachycardia, tachypnea, muffled heart sounds, gallop rhythm	Gene Xpert (sputum)	Multiple caseating granulomas (lymph node biopsy); Exudative pericardial fluid	Massive pericardial effusion with reduced LV function (EF 30%)	HRZE, prednisolone; furosemide *, spironolactone *, hydrochlorothiazide *, dapsone *	Emergency pericardiostomy (2 L of serosanguineous fluid over six weeks)	Positive
Taxak et al. [24] 2022 India	1	F 11 y	NR	Ascites, splenomegaly, hepatomegaly, dilation of IVC, SVC, and main pulmonary artery	NR	NR	“Restricted filling of hypocontractile LV”	ATT	Pericardiectomy	Positive
Mucheleng’anga et al. [25] 2022 Zambia	1	M 14 y	Absent	Absent	NP	Autoptic findings of TB pancarditis	NP	NP	NP	Negative
Swaminathan [26] 2020 Tajikistan	1	M 2.3 y	Fever, cough, weight loss, decreased activity	Absent	GenoType MTBDRplus, H-resistant MBT (pleural fluid)	NP	“Thickened pericardium with no effusion or calcifications”	HRZE; capreomycin ^†^, moxifloxacin ^†^, cycloserine ^†^, PAS ^†^, protionamide^†^, linezolid ^†^; prednisone	Pericardiectomy	Positive
Khera et al. [27] 2020 India	1	F 11 y	Fever, weight loss, loss of appetite, multiple mediastinal lymphadenopathy	Hepatomegaly, exertional dyspnea, lower limbs pitting edemas, facial edema, increased JVP, tachycardia, tachypnea, muffled heart sounds, gallop rhythm	Absent	NP	“Thickened pericardium; thickened myocardium with speckled calcifications, intracardiac mass on RA, biventricular dysfunction”	ATT; prednisolone; furosemide *, enalapril *	NP	Positive
Shah et al. [28] 2020 India	1	F 8 y	Fever, weight loss, pallor, calcified mediastinal lymph nodes encasing the aorta	Ascites	AFB (abscess sample smear)	NR	“Solidified collection in superior mediastinum encasing the aorta”	ATT, streptomycin ^†^, ethambutol ^†^, linezolid ^†^, amikacin^†^, levofloxacin ^†^, moxifloxacin ^†^, PAS ^†^, cyloserine ^†^, prednisolone	Pericardiocentesis	Positive
Shodikin et al. [62] 2020 Indonesia	1	M 14 y	Fever, low body weight, conjunctival anemia, anemia	Dyspnea, chest pain, distended abdomen, JV distention, tachycardia, tachypnea, distant heart sounds	NR	Hemorrhagic fluid with histiocytes and no granulomas	Massive pericardial effusion with preserved EF; low voltage QRS (ECG)	HRZE, methylprednisolone, pyridoxine, furosemide *	Pericardiocentesis and catheterization (1.2 L)	Positive
Paramitha et al. [20] 2020 Indonesia	1	M 12 y	Fever, fatigue, pallor, malnutrition	Dyspnea	AFB (pericardial fluid)	NP	Pericardial effusion	HRZE, ciprofloxacin *, ampicillin * and cloxacillin *; prednisone	Pericardiocentesis (300 mL of fluid)	Positive
	1	M 17 y	Cough, malnutrition, acute pharyngitis	Dyspnea	AFB (sputum) Gene Xpert (sputum) elevated ADA (pericardial fluid)	NP	“Massive pericardial effusion”	HRZE; corticosteroids	Emergency pericardiocentesis (1.15 L, repeated); Pericardial window	Positive
	1	F 17 y	Fever, weight loss, malnutrition, anemia, vomiting, abdominal pain	Dyspnea, hepatomegaly, ascites, tachycardia, tachypnea, muffled heart sounds	Elevated ADA (pericardial fluid)	NP	“Massive circumferential pericardial effusion, right ventricular and atrial collapse, swinging heart;” low voltage QRS (ECG)	ATT	Pericardiocentesis (460 mL xanthochromic fluid); Catheterization	Positive
Noguera-Julian et al. [29] 2020 NR	4	F (1) 12.1 y	Fever, cough, fatigue, anorexia	NR	Culture and PCR (sputum)	NP	NR	HRZE, ofloxacin, amikacin; steroids * and methotrexate *	NP	Positive
		M (1) 8.6 y	Fever, cough, headache, weight loss, joint pain	Ascites, abdominal distention	Culture and PCR (gastric aspirate)	NP	NR	HRZE; steroids	NP	Positive
		M (1) 16.1 y	Fever, cough, weight loss, night sweats	Dyspnea, chest pain	PCR (lung biopsy)	NP	NR	HRZE; clarithromycin, amikacin	NP	Positive
		F (1) 10.6 y	Fever, cough, weight loss, intestinal symptoms	NR	S-resistant MTB (gastric aspirate)	NP	NR	HRZE, quinolones ^†^; steroids	NP	Positive
Brotherton et al. [30] 2019 Kenya	1	M 5 y	Fever, cough, fatigue, loss of appetite, anemia, severe malnutrition	Dyspnea, chest pain, tachypnea, muffled heart sounds	Absent	NP	“Moderate pericardial effusion with extensive fibrinous exudate from pericardium to epicardium”	ATT (4 drugs); prednisone	NP	Positive
Martínez et al. [31] 2019 Argentina	1	NS 16 y	Absent	Hepatomegaly, ascites and abdominal distension, increased IVC, abdominal pain, and weight gain (due to hepatomegaly)	Culture (NR)	“Severely thickened, rigid, and adhered pericardium with myocardial akinesia, fibrosis and lymphocytic infiltration”	“Biauricular dilation, mitral valve prolapse, mild to moderate mitral regurgitation, dyskinetic interventricular septum; increased pulmonary pressure” (cardiac catheterization)	HRZE; furosemide *, carvedilol *, enalapri l *	Pericardiectomy	Positive
Kumar et al. [32] 2019 India	1	M 7 y	fever, emaciation, pallor	dyspnea, engorged neck veins, ascites, tense abdomen, muffled heart sounds	Elevated ADA (ascitic fluid)	“Thick, whitish and glistening pericardium”	“Thickened pericardium, paradoxical movement of interatrial septum”	ATT	anterior pericardiectomy	Positive
Obihara et al. [19] 2018 South Africa	30	F (12) M (18) 3.9 y (median)	Fever (13), cough (20), weight loss (25), night sweats (4), Abdominal pain (4), diarrhea (4), vomiting (4), lymphadenopathy (19), clubbing (4), rash (3), Peripheral lymphadenopathy (13)	Hepatomegaly (21), splenomegaly (6), dyspnea (8), chest pain (3), distended abdomen (10), malnutrition edemas (7), tachypnea (7), pericardial friction rub (3)	Culture (respiratory specimens) (19); Culture (lymph node aspirates) (6); Culture (pericardial fluid/tissue) (2); Culture (cerebrospinal fluid) (1) GenoType MTBDRplus (2 MDR, 1 RMR)	NR	Pericardial effusion (29)	ATT (30), second-line agents ^†^ (3); prednisone (24); diuretics * (3)	Pericardiocentesis (465 mL median) (5); Pericardiectomy (2)	Positive (20); Negative (1); NR (9)
Girit et al. [33] 2018 Turkey	1	F 15 y	Fever, cough	Severe exertional dyspnea, orthopnea, chest pain, JV distension, pulsus paradoxus, decreased heart sounds, tachycardia	PCR (pericardial fluid) elevated ADA (pericardial fluid)	Necrotic granulomatous reaction	Massive pericardial effusion and fibrin-coated mass adjacent to RV; low QRS voltage (ECG)	HRZE; corticosteroids	emergency pericardiocentesis (1 L of hemorrhagic fluid drained); partial pericardiectomy and abscess drainage (median sternotomy)	Positive
Dayal et al. [34] 2018 India	1	M 10 y	Absent	Anasarca (sole initial presentation), hepatomegaly, ascites (abdomen US), tachycardia, tachypnea	AFB (pericardial biopsy), elevated ADA (pericardial fluid)	“Dense adhesions between pericardium and pleural cavity, purulent fluid, multiple epithelioid granulomas”	Moderate pericardial effusion, organized pericardial collections	HRZE; steroids	Pericardiectomy; catheterization	Positive
Abreu Suárez et al. [35] 2018 Cuba	1	F 17 y	Fever, weight loss, subcarinal and mediastinal lymphadenopathy	Absent	Culture (biopsy of pre-sternal lesion)	NP	NR	ATT (5 drugs); steroids	Pericardial drainage	Positive
Igoche et al. [36] 2017 Nigeria	1	M 4 y	Fever, cough, weight loss, generalized lymphadenopathy	Hepatomegaly increased JVP, distended neck veins, tachypnea, tachycardia, distant heart sounds	Absent	NP	Massive circumferential pericardial effusion, heart swinging, RA and RV collapse	ATT	Pericardiocentesis (180 mL of creamy pus), Pericardiostomy	Positive
Jakimów-Kostrzewa [37] 2017 Poland	1	F 1.5 y	High fever, cough, mediastinal and hiatal lymphadenomegaly	Dyspnea	“Microbiological and genetic analysis”	NP	“Banana-like shaped encapsulated mass”	ATT	Surgical removal of the mass	Positive
Melit et al. [38] 2017 Romania	1	M 15 y	Fever, cough, malaise, rhinorrhea, saburral tongue, hyperemic pharynx, hypertrophic tonsils, dizziness, vomiting	Orthopnea, thoracic pain, bleared cardiac sounds, arterial hypotension	PCR (pericardial fluid)	Aspecific evidence of inflammatory cells	Pericardial effusion	ATT; furosemide *, spironolactone *; cephalosporin *, aminoglycoside *, meropenem *, vancomycin *, fluoroquinolone *	Pericardiocentesis (150 mL of serohemorrhagic fluid)	Positive
Chiu et al. [39] 2016 Taiwan	1	M 4 y	Cough	Dyspnea, hepatomegaly, JV engorgement, tachycardia, tachypnea, subcostal retractions	Culture (pericardial surgical specimen)	Caseous necrosis (surgical specimen)	Dip and plateau pattern confirmed with cardiac catheterization (constrictive pericarditis)	ATT; corticosteroids	Pericardiectomy	Positive
Faustino et al. [40] 2015 Portugal	1	M 14 y	Low body weight	Hepatomegaly, ascites, congestion of JV, tachypnea	Culture (sputum) (11 y)	Thickened, adherent, non-calcified pericardium with extensive fibrosis	Enlargement of RA, RV, IVC, and suprahepatic veins, septal bounce; “constrictive pericarditis suspicion confirmed with cardiac catheterization”	ATT; diuretics *	Pericardiectomy	Positive
Yoon et al. [41] 2012 South Korea	1	M 14 y	Fever, cough, malaise, night sweats, bilateral hilar lymphadenopathy	Tachycardia, tachypnea	AFB H-resistant (sputum) AFB (pericardial fluid) AFB (biopsy of intrapericardial masses) PCR (biopsy of intrapericardial masses)	“Soft yellowish discoid masses composed of pink, amorphous meshwork of threads”, RBC, WBC	“Free-floating multiple round discoid masses in a large amount of pericardial effusion”	HRZE, prednisone	Pericardiostomy (removal of masses)	Positive
Gupta et al. [42] 2012 India	1	M 1.5 y	Fever, pallor	Hepatomegaly, facial edema, ascites, tachycardia	Absent	“Organizing fibrinous pericarditis” (surgical specimen)	“Mild, organized pericardial effusion and thickened pericardium”; findings consistent with TOF	ATT	Pericardiectomy	Positive
Campagnucci et al. [43] 2012 Brazil	1	F 10 y	Joint pain	NR	Absent	Diffuse fibrous bands form tight adhesions. Anterior descending coronary artery compressed by fibrous bands	Pericardial effusion	ATT	Pericardiocentesis, surgical removal of mediastinal adhesions	Positive
Gulati et al. [44] 2011 India	1	M 12 y	Absent	Exertional fatigue, hepatomegaly, raised JVP, tachycardia	NR	NR	“Infiltrating mass lesion involving RV and LV; Incomplete right bundle branch block and prominent, diffuse ST-T changes, saddle-shaped ST elevation in V1” (ECG)	ATT	NP	Positive
Rabie et al. [45] 2010 South Africa	1	M 3 y	Fever, anemia	Abdominal distention due to ascites	AFB (bone marrow aspirate); Culture (bone marrow aspirate); Culture (blood)	Granulomas (bone marrow aspirate)	“Pericardial effusion with diastolic dysfunction”	HRZE, ethionamide, prednisone, methylprednisolone	NP	Positive
Takawira et al. [46] 2010 South Africa	1	M 3 y	Fever, cough, night sweats, underweight, generalized lymphadenopathy (axillary, supraclavicular, cervical, submandibular, paratracheal)	Hepatomegaly, splenomegaly, generalized body edemas, ascites, tachypnea, tachycardia, muffled heart sounds, gallop rhythm	AFB (lymph node biopsy); Culture (gastric aspirate)	Extensive fibrosis in the thoracic cavity and pericardial space (surgery); caseating granulomas (biopsy of supraclavicular lymph node)	“Loculated pericardial effusion”	HRZE; prednisone; cardiac failure treatment *	Surgery for the subaortic aneurysm	Positive
Lee et al. [47] 2010 South Korea	1	M 14 y	Fever, cough, weakness, pallor	Exertional dyspnea, hepatomegaly, tachycardia, tachypnea, systolic murmur, friction rub (after pericardiocentesis), weak peripheral pulses	Elevated ADA (pericardial fluid)	NR	“Large pericardial effusion and diastolic collapse of RV wall”; low QRS voltage and flat T waves (ECG)	NR	Emergency pericardiocentesis	Positive
Massoure et al. [61] 2010 Djibouti	1	M 16 y	NR	Cardiac tamponade	Culture (pericardial fluid), after treatment initiation	Pericardial fibrinous pockets, fibrinous strands	Thick echogenic porridge-like pericardial effusion, compression of RV and RA, IVC 20 mm; ST elevations (ECG)	HRZE	Pericardiocentesis (serosanguineous fluid), pericardiotomy and catheterization	Positive
El Samady et al. [48] 2009 Saudi Arabia	1	F 7 y	Fever, cough, weight loss	Absent	NR	NP	Pericardial calcification	ATT	NP	NR
Bolt et al. [60] 2007 Netherlands	1	F 15 y	Fever, cough, weight loss, decreased appetite, fatigue, anemia	Dyspnea, chest pain, hepatomegaly, soft cardiac tones, friction rub, tachypnea, hypotension	PCR (pericardial biopsy)	NR	“Large pericardial effusion with exudative debris and fibrin strands”	HRZE; prednisolone	Pericardiocentesis	Positive
Çetin et al. [49] 2005 Turkey	1	M 8 y	Absent, abdominal pain, arthralgia	Massive hepatosplenomegaly, anasarca, ascites, distended neck veins, dilated IVC, pulsus paradoxus, tachycardia, gallop rhythm	Absent	Fibrinous pericarditis; pericardium infiltrated with lymphoid aggregates, fibrin, and capillary proliferation (autoptic finding)	“Four-sided pericardial effusion, massively thickened pericardial wall, massive dilatation of RA and RV”	ATT (2 drugs); diuretic and positive inotropic therapy *	Pericardiectomy	Negative
Sharifi-Mood et al. [50] 2005 Iran	1	F 6 y	Fever, loss of appetite	Exertional dyspnea, tachycardia, tachypnea	Absent	“Cyst in the mediastinal area extending to pericardium; necrosis with caseating granuloma”	Nonspecific ST segment and T-wave changes (ECG)	HRZE; steroids	Surgical partial removal of the cyst	Positive
Meyburg et al. [51] 2002 Germany	1	F 2.5 y	Fever, cough	Hepatomegaly, dilation of IVC, tachypnea, tachycardia	AFB (pericardial fluid); PCR (pericardial fluid); Culture (pericardial fluid) PCR (gastric aspirate)	NP	Large pericardial effusion and dilation of IVC	HRZS; prednisone	Emergency pericardiocentesis (220 mL of thick amber fluid, repeated)	Positive
Browne et al. [52] 2002 Australia	1	M 14 y	Fever	Respiratory distress, chest pain, enlarged JV, pericardial rub	AFB (sputum and pericardial fluid)	Fibrosis	“Large effusion, persistent with some adherence”	HRZ; prednisone	Pericardiocentesis (hemoserous fluid 250 mL); catheterization; pericardiectomy	Positive
Tutar et al. [53] 2002 Turkey	1	NR 4 y	NR	“Signs and symptoms of constrictive pericarditis”	NR	*Histological diagnosis of TBP*	Findings indicative of constrictive pericarditis	ATT	Surgical drainage of effusion, pericardiectomy	NR
Equi et al. [54] 2001 UK	1	F 10 y	Cough, lethargy, weight loss, extensive mediastinal lymphadenopathy with some calcification	Chest pain	PCR (lymph node biopsy) INNO-LiPA Rif.TB (lymph node biopsy)	Granuloma with necrosis and calcification (lymph node biopsy)	“Global pericardial effusion”	HRZ, pyridoxine	Pericardiocentesis (700 mL bloodstained fluid); Pulmonary endarterectomy and autologous pericardial patch repair	Positive
Maltezou et al. [21] 2000 Greece	1	NR 9 y	Fever, weight loss	Dyspnea, respiratory distress, chest pain, ascites	Culture (pericardial fluid) Culture (gastric aspirate)	NP	NR	HRZS; corticosteroids	Pericardiocentesis	Positive
Lin et al. [55] 2000 Taiwan	1	F 0.8 y	Fever, poor appetite	Hepatomegaly, edema	NR	“Tuberculous fibrinofibrous pericarditis”	“Solid mass originating from the thickened pericardium, compressing the heart”	ATT	Pericardiectomy	Positive
Weber et al. [56] 1999 Zimbabwe	4	F (1) 6 y	Cough and dysentery	Absent	Culture (gastric washing)	NP	Pericardial effusion	ATT	NP	Positive
		M (1) 11 y	Unspecific complaints	Absent	Culture (gastric washing)	NP	Pericardial effusion	ATT	NP	Positive
		M (1) 0.2 y	Meningitis	Absent	Culture (gastric washing)	NP	Pericardial effusion	ATT	NP	Negative
		M (1) 0.9 y	Failure to thrive intestinal obstruction	Absent	Culture (gastric washing)	NP	Pericardial effusion	NP	NP	Negative
Coulter et al. [11] 1996 UK	1	M 14 y	Fever, anorexia, weight loss, weakness, anemia, lymphadenomegaly (mediastinal and pretracheal)	Chest pain, hepatomegaly, tachypnea, decreased exercise tolerance, pulsus paradoxus, increased JVP, quiet heart sounds, tachycardia	Culture (pericardial aspirate, sputum); AFB (sputum)	NP	Large pericardial effusion with atrial compression, pericardial thickening, poor LV function, and mild tricuspid regurgitation; widespread T-wave inversion (ECG)	HRZ; prednisolone; blood transfusions	Pericardiocentesis (1 L of serosanguineous fluid, repeated with 1.15 L)	Positive
Cohen et al. [57] 1995 US	1	F 12 y	Fever, anemia	Chest pain	Absent	Caseating granulomas (mediastinal mass biopsy)	Pericardial effusion	HRZ	Pericardiocentesis (180 mL of serosanguineous fluid)	Positive
Nelson et al. [58] 1995 US	1	M 16 y	Fever, lymphadenopathy, sore throat	Dyspnea, chest pain, hepatomegaly	Fluorescence microscopy (pericardial biopsy); AFB kinyoun (pericardial biopsy); PCR (pericardial fluid)	Thickened pericardium with fibrinous exudate	*Large pericardial effusion with fibrinous bands; * concave ST elevation, decreased QRS (ECG)	HRZS; methylprednisolone	Pericardiocentesis (400 mL), pericardial window	Positive
Hugo-Hamman et al. [18] 1994 South Africa	44	F (24), M (20) 3.75 y (median)	Fever (28), cough (31), weight loss (16), Abdominal pain (5), vomiting (3), anemia (32)	Dyspnea (34), chest pain (13), hepatomegaly (34), elevated JVP (34), pulmonary edema (9), edema (7), pulsus paradoxus (18), friction rub (8)	Culture (pericardial fluid) (1); AFB (pericardial fluid) (1); Culture (pericardial biopsy) (1); AFB (pericardial biopsy) (1); AFB (gastric aspirate) (2); AFB (lymph node biopsy) (2) elevated ADA (pericardial fluid) (14)	Histologic features of MTB on pericardial biopsy (1)	Indicative signs of pericarditis (44), variable size effusion (37), thickened echo-bright pericardium (4), small effusion with a thick caseous coating (3); Complete ECG (28), low voltage QRS (7), diffuse T wave inversion (8), ST changes (3)	HRZ (44), E (39), prednisolone (18)	Pericardiectomy (5), pericardiocentesis (20), pericardial window (14)	Positive (43), Negative (1)
Kher et al. [59] 1990 India	1	F 6.5 y	Fever	Dyspnea, hepatomegaly, splenomegaly, lower limbs edemas, ascites, increased JVP, tachycardia, decreased heart sounds	‘TB antigen’ (pericardial fluid)	Chronic inflammation (biopsy)	Low voltage QRS and elevated ST segments (ECG)	ATT	Pericardiocentesis (hemorrhagic fluid)	Positive

*: medications used to treat heart failure or conditions other than TBP; †: second-line antitubercular treatment for resistant MTB.

**Table 2:** Main reported signs and symptoms and their relative frequency.

Relevant Reported Signs and Symptoms	Number of Patients	Frequency
fever	76	62%
hepatomegaly	74	60%
cough	72	59%
dyspnea	64	52%
weight loss or low body weight	60	49%
increased JVP or JV turgor	46	37%
chest pain	28	23%
lymphadenopathy	24	20%
tachypnea	23	19%
edema	22	18%
pulsus paradoxus	21	17%
tachycardia	17	14%
abdominal distention	16	13%
ascites	15	12%
muffled heart sounds	13	11%
friction rub	13	11%
abdominal pain	12	10%
splenomegaly	10	8%
pulmonary edema or orthopnea	10	8%
vomiting	9	7%
night sweats	8	7%
fatigue	9	7%
gallop rhythm	4	3%

**Table 3:** Bodily sites affected by TB other than the pericardium.

Other TB Manifestations	Number of Cases	Frequency
Pulmonary TB	53	42%
Pleural effusion	46	37%
Lymphadenopathy	37	30%
Disseminated or miliary TB	19	15%
Abdominal TB	18	14%
TB Meningitis	4	3%
Cardiac TB	4	3%
TB Abscesses	3	2%

## Data Availability

No new data were created or analyzed in this study. Data sharing is not applicable to this article. The systematic review registration number is CRD42023482728.

## Supplementary Materials

The following supporting information can be downloaded at: www.mdpi.com/xxx/s1,additional file S1: search strategy; additional file S2: definition of TBP diagnosis; additional file S3: variables included; additional file S4: list of countries; additional file S5: list of comorbidities.

## Abbreviations

AFB	Acid-fast bacilli
ADA	Adenosine deaminase
AF	Antitubercular treatment
ART	Antiretroviral therapy
ATT	Antitubercular treatment
CXR	Chest X-ray
ECG	Electrocardiogram
EPTB	Extrapulmonary tuberculosis
Genotype MTBDRplus	
HAART	Highly active antiretroviral therapy
HRZE	Isoniazid, rifampicin, pyrazinamide, ethambutol
IRIS	Immune Reconstitution Inflammatory Syndrome
IVC	Inferior vena cava
JIA	Juvenile Idiopathic Arthritis
JVP	Jugular venous pressure
LABD	Linear IgA Bullous Dermatitis
LA	Left atrium
LV	Left ventricle
MTB	Mycobacterium tuberculosis
NP	Not performed
NR	Not reported
PCR	Polymerase chain reaction
PAS	Para-aminosalicylic acid
RA	Right atrium
RV	Right ventricle
SVC	Superior vena cava
TB	Tuberculosis
TBP	Tuberculous pericarditis
