# Supplementary material for: Tuberculous Pericarditis in Childhood: A Case Report and a Systematic Literature Review
Source: Pathogens. 2024 Jan 26;13(2):110. doi: 10.3390/pathogens13020110 (PMC10892678; doi:10.3390/pathogens13020110)
Supplement: Supplementary file 1 [file pathogens-13-00110-s001.zip › Additional file 1_search strategy.pdf]

Additional file 1 – search strategy

PUBMED

(("tuberculous pericarditis"[Text Word] OR "pericarditis, tuberculous"[MeSH Terms] OR (("tubercul*"[tiab] OR "tuberculosis"[MeSH Terms]) AND ("pericard*"[tiab] OR "pericarditis"[MeSH Terms] OR "pericardial effusion"[MeSH Terms] OR "pericardial fluid"[MeSH Terms]))) AND ("child"[tiab] OR "children"[tiab] OR "infant*"[tiab] OR "newborn"[tiab] OR "child"[MeSH Terms] OR "infant"[MeSH Terms] OR "pediatrics"[MeSH Terms] OR "pediatric*"[tiab] OR "paediatric*"[tiab])) AND (1990:2023[pdat])

SCOPUS

TITLE-ABS-KEY({tuberculous pericarditis} OR ("tubercul*" AND "pericard*") AND TITLE-ABS-KEY ({child} OR {children} OR “infant*" OR {newborn} OR "pediatric*" OR "paediatric*") AND (PUBYEAR > 1989 AND PUBYEAR < 2024)
