# Supplementary material for: Tuberculous Pericarditis in Childhood: A Case Report and a Systematic Literature Review
Source: Pathogens. 2024 Jan 26;13(2):110. doi: 10.3390/pathogens13020110 (PMC10892678; doi:10.3390/pathogens13020110)
Supplement: Supplementary file 1 [file pathogens-13-00110-s001.zip › Additional file 2_Definition of TBP Diagnosis.pdf]

Additional file 2 - Definition of tuberculous pericarditis diagnosis

For the purpose of this systematic review, cases of tuberculous pericarditis (TBP) were categorized as either "certain" or "probable" based on the following criteria. A "certain" diagnosis is established when there is confirmed evidence of Mycobacterium tuberculosis (MTB) presence in the pericardium (fluid or tissue), and/or histological findings consistent with tuberculous infection (such as granulomas or chronic inflammatory infiltrates compatible with MTB infection). A "probable" diagnosis of TBP is assigned when pericarditis is detected along with MTB evidence in other anatomical sites, elevated adenosine deaminase (ADA) levels in pericardial fluid, or epidemiological and clinical characteristics indicative of tuberculosis, or when there is clinical improvement upon receiving anti-tuberculosis treatment (ex juvantibus criterion).
