# Supplementary material for: Tuberculous Pericarditis in Childhood: A Case Report and a Systematic Literature Review
Source: Pathogens. 2024 Jan 26;13(2):110. doi: 10.3390/pathogens13020110 (PMC10892678; doi:10.3390/pathogens13020110)
Supplement: Supplementary file 1 [file pathogens-13-00110-s001.zip › Additional file 3_variables included.pdf]

Additional file 3 - Variables included in Excel spreadsheets

For every included paper, we extracted the following information: name of first author, year of publication, country where the study was conducted, number of patients, age (absolute number) or median age, gender of the patient, signs and symptoms, TBP subtype, other bodily site affected by TB, comorbidities, type and sample used for the reported microbiological tests with positive outcome, ADA levels considered elevated by the authors, type and sample used for the reported microbiological test with negative outcome, outcome of TST or IGRA tests, histological reports, echocardiographic and ECG findings, imaging findings (CT or CXR), medical treatment, surgical treatment, outcome.
