# Supplementary material for: Tuberculous Pericarditis in Childhood: A Case Report and a Systematic Literature Review
Source: Pathogens. 2024 Jan 26;13(2):110. doi: 10.3390/pathogens13020110 (PMC10892678; doi:10.3390/pathogens13020110)
Supplement: Supplementary file 1 [file pathogens-13-00110-s001.zip › Additional file 4_List of countries.pdf]

**Additional file 4 - List of countries where the collected studies were conducted**

1. Argentina (Buenos Aires)
2. Australia (Sydney)
3. Brazil (São Paulo)
4. Cuba (Havana)
5. Djibouti (Djibouti)
6. Germany (Heidelberg)
7. Greece (Athens)
8. India (Mumbai, Chandigarh, New Delhi)
9. Indonesia (Jember, Yogyakarta)
10. Iran (Zahedan)
11. Kenya (Kijabe)
12. Netherlands (Amsterdam)
13. Nigeria (Kano, Kwara)
14. USA (Philadelphia, Pennsylvania; Houston, Texas)
15. Poland (Warsaw)
16. Portugal (Lisbon)
17. Romania (Târgu Mureș)
18. Saudi Arabia (Riyadh)
19. South Africa (Cape Town, Pretoria)
20. South Korea (Cheongju, Seongnam)
21. Tajikistan (Dushanbe)
22. Taiwan (Taipei)
23. Turkey (Ankara, Istanbul)
24. UK (Liverpool, London)
25. Zambia (Lusaka)
26. Zimbabwe (Gweru)
