# Supplementary material for: Tuberculous Pericarditis in Childhood: A Case Report and a Systematic Literature Review
Source: Pathogens. 2024 Jan 26;13(2):110. doi: 10.3390/pathogens13020110 (PMC10892678; doi:10.3390/pathogens13020110)
Supplement: Supplementary file 1 [file pathogens-13-00110-s001.zip › Additional file 5_comorbidities.pdf]

Additional file 2 - List of comorbidities observed

1. One patient had linear IgA bullous dermatosis (LABD)
2. One patient had non-tubercular bilateral pneumonia and situs viscerus inversus
3. One patient had pneumonia caused by Enterobacter cloacae and Candida tropicalis
4. One patient had cystitis
5. One patient had systemic Juvenile Idiopathic Arthritis (JIA) and probable immune reconstitution inflammatory syndrome (IRIS)
6. One patient had polyarticular JIA
7. One patient had Chron's disease and primary sclerosing cholangitis
8. One patient had ulcerative colitis
9. 9 patients were HIV positive, one of them also had IRIS
10. One patient had acute osteomyelitis (S. Aureus) and pyopericardium
11. One patient had interstitial pneumonia
12. One patient had tetralogy of Fallot
13. One patient was HIV-positive, had IRIS, chylous ascites and chylothorax
14. One patient had Takotsubo syndrome
15. One patient had protein-losing enteropathy and two patients had Takayasu disease
16. One patient had congenital lypodistrophy
17. One patient had a hydatid cyst localized in the pericardium
18. One patient had Arrhythmogenic right ventricular dysplasia (ARVD)
